# Bacterial Expression, Purification and *In Vitro* Phosphorylation of Full-Length Ribosomal S6 Kinase 2 (RSK2)

**DOI:** 10.1371/journal.pone.0164343

**Published:** 2016-10-12

**Authors:** Darkhan Utepbergenov, Paulina M. Hennig, Urszula Derewenda, Mykhaylo V. Artamonov, Avril V. Somlyo, Zygmunt S. Derewenda

**Affiliations:** 1 Department of Molecular Physiology and Biological Physics, University of Virginia, School of Medicine, Charlottesville, Virginia, United States of America; 2 Department of Molecular Genetics, University of Lodz, Lodz, Poland; Queen Mary University of London, UNITED KINGDOM

## Abstract

Ribosomal S6 kinases (RSK) play important roles in cell signaling through the mitogen-activated protein kinase (MAPK) pathway. Each of the four RSK isoforms (RSK1-4) is a single polypeptide chain containing two kinase domains connected by a linker sequence with regulatory phosphorylation sites. Here, we demonstrate that full-length RSK2—which is implicated in several types of cancer, and which is linked to the genetic Coffin-Lowry syndrome—can be overexpressed with high yields in *Escherichia coli* as a fusion with maltose binding protein (MBP), and can be purified to homogeneity after proteolytic removal of MBP by affinity and size-exclusion chromatography. The purified protein can be fully activated *in vitro* by phosphorylation with protein kinases ERK2 and PDK1. Compared to full-length RSK2 purified from insect host cells, the bacterially expressed and phosphorylated murine RSK2 shows the same levels of catalytic activity after phosphorylation, and sensitivity to inhibition by RSK-specific inhibitor SL0101. Interestingly, we detect low levels of phosphorylation in the nascent RSK2 on Ser386, owing to autocatalysis by the C-terminal domain, independent of ERK. This observation has implications for *in vivo* signaling, as it suggests that full activation of RSK2 by PDK1 alone is possible, circumventing at least in some cases the requirement for ERK.

## Introduction

**Fig 1:**
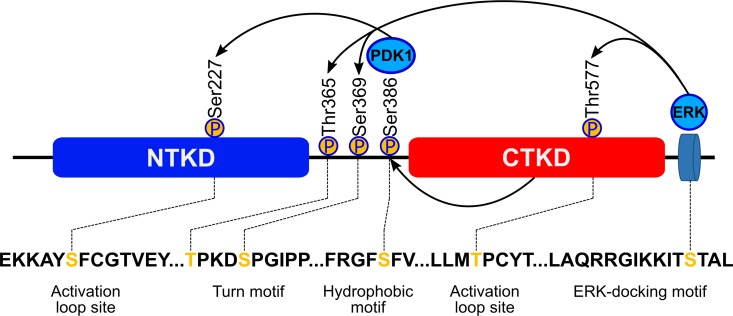
Structural organization of RSK2 and the canonical scheme of the activating phosphorylation cascade.

The four isoforms of the ribosomal S6 p90 protein kinase (RSK1-4), along with the two closely related isoforms of the mitogen- and stress-activated protein kinase (MSK1-2), constitute a unique family of Ser/Thr kinases, which are made up of single polypeptide chains harboring two Ser/Thr kinase catalytic domains in tandem [1–5]. All these enzymes mediate signaling downstream of the mitogen-activated protein kinases (MAPKs) which include, among others, the ERK, JNK and p38 kinases, and regulate cell proliferation, gene expression, mitosis, apoptosis, muscle contraction, differentiation and a range of other cellular functions [6, 7]. Both RSKs and MSKs are activated through regulatory phosphorylation by kinases of the MAPK pathway and subsequently transmit the signal downstream by phosphorylating specific proteins. The activation mechanism is complex, owing to the unique architecture of RSKs and MSKs **(Fig 1)**. There are two catalytic domains: the N-terminal kinase domain (NTKD), which belongs to the AGC family and which is the biologically active module that phosphorylates downstream protein targets; and the C-terminal kinase domain (CTKD), with homology to the calmodulin-dependent family [1, 2, 4], involved in autoregulation of the enzyme. The two modules are connected by a ~70 amino acid regulatory linker, which harbors phosphorylation sites, specifically within the so-called turn and hydrophobic motifs [8, 9]. The current model of the activation process of these kinases, involves several trans- and cis-phosphorylation steps. In RSK, ERK1/2 docks at the C-terminus and phosphorylates the activation loop in CTKD (Thr577 in RSK2), thereby conferring catalytic activity on that domain. It also phosphorylates two additional sites within the linker (Thr365 and Ser369 in RSK2). The activated CTKD then phosphorylates a serine within the so-called hydrophobic motif (Ser386 in RSK2), creating a docking site for the phosphoinositide-dependent kinase 1 (PDK1). The latter phosphorylates the activation loop in NTKD (Ser227 in RSK2) conferring full biological activity on RSK.

Recently, there has been a surge in interest in the molecular physiology and inhibitor design for the RSK kinases, and particularly for RSK2. This is because the level of RSK2 expression and phosphorylation is significantly higher in a subset of MAPK driven cancer cell lines as compared to non-cancer controls, and RSK2 is therefore considered to be a viable cancer drug target [10–13], specifically in the treatment of breast [14] and prostate tumors [15–17], myeloma [18, 19], T-cell lymphoma [20] and melanoma [21].

RSK2 is also involved in a hematopoietic transformation: when compared to the wild type, knockout mice lacking RSK2 showed much higher survival rate upon induction of myeloma by transplantation of oncogenic bone marrow [22]. Similarly, studies of skin cancer [23] and c-Fos dependent osteosarcoma [24] indicate an important role of RSK2 in neoplastic transformation.

Cancer is not the only pathological state in which RSK kinases play a part. Mutations in the gene coding for RSK2 have been associated with the Coffin-Lowry Syndrome [25]. Another member of RSK family, RSK1, has been shown to mediate pathological effects of ischemia-reperfusion *via* phosphorylation of the Na^+^/H^+^ exchanger isoform 1 both in the heart and in brain [26–29]. RSKs have also been implicated as mediators of the increased Na^+^/H^+^ exchange activity in vascular smooth muscle found in hypertension, based on the ability of angiotensin II to stimulate RSK in an ERK and Ca2+-dependent fashion and activate this exchanger [30]. Thus, selective inhibition of RSK isoforms may help, for example, in the treatment of post myocardial infarction injury to the heart for which there are no drugs at all [31–35] and in cases of difficult to treat hypertension [36].

Biophysical studies of the RSK kinases and structure-based drug discovery have been limited by the difficulties in the preparation of homogeneous and pure samples of recombinant protein. Structural studies focused on the isolated NTKD and CTKD domains, which can be produced in *E*. *coli*. However, these studies were not able to reveal details of any conformational changes that in the full-length protein would be associated with the complex phosphorylation cascade and activation process. The full-length RSK kinases are typically overexpressed in eukaryotic systems, often in insect cells, with low yield and at considerable cost. Moreover, these recombinant proteins are already phosphorylated *in situ* and active. Although expression of full-length, His-tagged RSK2 in *E*. *coli* has been reported, little or no information has been provided about purification procedures, yield, purity or specific activity [19, 37]. Here we report a new protocol for expression and purification of the full-length, 740 residue-long murine RSK2 in *E*. *coli*, utilizing MBP as a fusion protein to boost expression yield and a His_6_-tag to facilitate purification. Using this recombinant protein, mass spectrometry and western blots, we were able to confirm the canonical activation mechanism, although we discovered that the CTKD of RSK2 is able to phosphorylate Ser386 in the absence of ERK1/2 (although at a much slower rate), thus making it possible for PDK1 to activate NTKD independently of ERK1/2. Ser386 is the only site partly phosphorylated in the nascent enzyme, and it can be readily and efficiently dephosphorylated using Mn^2+^ dependent Lambda Protein Phosphatase (Lambda PP).

## Materials and Methods

### Antibodies and enzymes

Rabbit polyclonal antibodies raised against pSer227, pThr365/pSer369, and pThr577 of human RSK2 were purchased from Santa Cruz Biotechnology Inc. Rabbit monoclonal antibodies produced against pSer386 of human RSK-2 were purchased from Cell Signaling Technology. RSK-2 (E-1) mouse monoclonal IgG were purchased also from Santa Cruz Biotechnology Inc.

Fluorophore-labeled secondary antibodies Alexa Fluor® 680 goat anti-rabbit IgG (H+L) and IRDye®800CW Goat anti-Mouse were purchased respectively from Life Technologies™ and LI-COR. Lambda Protein Phosphatase was procured from New England BioLabs® Inc. RSK2 kinase from Sf9 cells and ADP-Glo kinase assay were purchased from Promega. Active PDK1 was purchased from EMD Millipore and active ERK2 was expressed and purified as described elsewhere [38].

### Expression and purification of RSK2

Full-length mouse RSK2 (amino acids 1–740, Genbank ID: **AY083469.1**) was amplified from mouse brain cDNA prepared in our laboratory (forward primer: AAAAAGGATCCATGCCGCTGGCGCAGCTGGCGGAC; reverse primer: AAAAAGTCGACTCACAGGGCTGTTGAGGTGATTTTTTTA) and cloned into pMBPHisParallel2 and pHisParallel2 vectors [39] using BamHI and SalI restriction sites. The constructs were verified by sequencing of purified plasmid. BL21(DE3) RIPL cells were transformed with a RSK2 expression constructs and plated on LB agar plates containing 100 μg/ml ampicillin and 34 μg/ml chloramphenicol. Transformed cells were transferred into 2 liters of terrific broth (TB) media and grown at 37°C to OD_600_~4.0. Thereafter the temperature was lowered to 16°C, expression was induced by the addition of isopropyl β-d-1-thiogalactopyranoside to 0.3 mM after which the cultures were grown for a further 16 hrs; cells were harvested by centrifugation and the cell pellets frozen at −80°C. For purification, cell pellets were thawed, re-suspended in 100 ml of lysis buffer (50 mM Tris pH 8.0, 500 mM NaCl, 10 mM imidazole, 4 mU/ml benzonase, 1 mM of freshly prepared PMSF, and 2 tablets of dissolved Complete® protease inhibitors), and lysed with EmulsiFlex-C3 homogenizer. Polyethylene imine was added to the lysate to a final concentration of 0.2% and the lysate was centrifuged at 80,000xg for 35 min. Clarified lysate was loaded onto 5 ml Ni-NTA column (HisTrap HP, GE Life Sciences), washed with lysis buffer without inhibitors and benzonase and eluted with a linear gradient of imidazole. Fractions containing RSK2 were pooled together and applied on a 4 ml gravity-flow amylose column, which was subsequently washed with Buffer A (20 mM Tris, 500 mM NaCl, pH 8.0). Proteins were eluted into the same buffer containing 10 mM maltose and immediately applied onto a 3 ml gravity-flow Ni-NTA column. The column was washed with 100 ml of Buffer A to remove maltose and bound proteins were eluted with Buffer A containing 250 mM imidazole. To cut off the MBP-(His)_6_ tag TEV protease has been added to a final concentration of 0.05 mg/ml and samples were dialyzed overnight against buffer A. To separate the MBP-(His)_6_ tag protein samples were passed through the 4 ml gravity-flow amylose column and flow-through fractions were pooled together, concentrated, and purified on Sephadex 200 size exclusion column equilibrated with Buffer A.

### RSK2 *in vitro* phosphorylation, immunoblot analysis and activity assay

For activation of RSK2, 4.8 μM samples of RSK2 were incubated in 0.05 μM of PDK1 and 0.047 μM of ERK (final concentration) in 20 mM Tris buffer containing 500 mM NaCl, 10 mM of MgCl2, and 200 μM of ATP at room temperature for 2 hrs. Aliquots of activation reaction were taken at different time points and analyzed for site-specific phosphorylation by immunoblot analysis. (Phosphorylation by either PDK1 or ERK alone, reported in Supplementary data was carried out in the same fashion).

Samples (~50 ng) of RSK2 were resolved on polyacrylamide gels, transferred to Immobilion R-FL polyvinylidine difluoride membrane (Millipore), which were then blocked with the Odyssey Blocking Buffer (TBS) LI-COR. The membranes were subjected to phospho-RSK2 (Ser 227, Ser 386, Thr 365/Ser 369, Thr 577) and total RSK-2 antibodies diluted in blocking odyssey buffer (1:8000). Next the membranes were washed 3 times in TBS with 0.05% Tween 20. Primary antibodies were visualized using fluorophore-labeled secondary antibodies conjugated to either Alexa Fluor® 680 or IRDye®800CW, and scanned with an Odyssey infrared imaging system (LI-COR Biosciences).

Activated RSK2 was assessed for enzymatic activity at room temperature using 0.2 mg/ml (160 μM) of S6 peptide and either 10 or 100 μM of ATP in a kinase buffer (40 mM Tris, 20 mM MgCl_2_). The enzymatic activity of RSK2 was determined by measuring the amount of ATP consumed in the reaction using ADP-Glo system (Life Technologies) and a luminescence plate reader (PHERAstar FS, BMG Labtech). In some experiments SL0101 at concentrations ranging between 0.1 and 100 μM was added to the reaction.

### Mass spectrometry

Protein bands were cut from the gels and transferred to a siliconized tube and washed in 200 μL 50% methanol. The gel pieces were dehydrated in acetonitrile, rehydrated in 30 μL of 10 mM dithiolthreitol in 0.1 M ammonium bicarbonate and reduced at room temperature for 0.5 h. The DTT solution was removed and the sample alkylated in 30 μL 50 mM iodoacetamide in 0.1 M ammonium bicarbonate at room temperature for 0.5 h. The reagent was removed and the gel pieces dehydrated in 100 μL acetonitrile. The acetonitrile was removed and the gel pieces rehydrated in 100 μL 0.1 M ammonium bicarbonate. The pieces were dehydrated in 100 μL acetonitrile, the acetonitrile removed and the pieces completely dried by vacuum centrifugation. The gel pieces were rehydrated in 20 ng/μL (trypsin, chymotrypsin, Glu-C, Asp-N and termolysin) in 50 mM ammonium bicarbonate on ice for 30 min. Any excess enzyme solution was removed and 20 μL 50 mM ammonium bicarbonate added. The sample was digested overnight at 37°C and the peptides formed extracted from the polyacrylamide in a 100 μL aliquot of 50% acetonitrile/5% formic acid. This extract was evaporated to 15 μL for MS analysis.

The LC-MS system consisted of a ThermoFisher Velos Orbitrap ETD mass spectrometer system with a Protana nanospray ion source interfaced to a self-packed 8 cm x 75 μm id Phenomenex Jupiter 10 μm C18 reversed-phase capillary column. 5 μL of the extract was injected and the peptides eluted from the column by an acetonitrile/0.1 M acetic acid gradient at a flow rate of 0.5 μL/min over 1.3 hours. The nanospray ion source was operated at 2.5 kV. The digest was analyzed using the rapid switching capability of the instrument acquiring a full scan mass spectrum to determine peptide molecular weights followed by product ion spectra (20) to determine amino acid sequence in sequential scans. This mode of analysis produces approximately 40000 MS/MS spectra of ions ranging in abundance over several orders of magnitude. The data were analyzed by database searching using the Sequest search algorithm.

### Biophysical characterization

The melting temperature (T_m_) of protein samples was determined using a fluorescence-based assay by monitoring the fluorescence intensity of SYPRO^®^Orange dye in the presence of the protein as a function of temperature. RSK2 at 0.2 mg/ml (final concentration) was mixed with 1x SYPRO^®^Orange Protein Gel Stain (Life Technologies) in 50mM Tris pH.8.0, 500mM NaCl, 5mM 2-mercaptoethanol. The thermostability experiments were carried out in StepOnePlus™ Real-Time PCR System (Applied Biosystems) with a temperature gradient from 20 to 90°C in the reaction volume of 10 μl. The first derivative of the resultant curve was calculated in order to determine the melting temperature (T_m_) for both nascent and activated RSK2. In separate experiments 40x stock solutions of the RSK inhibitors, BI-D1870 and SL0101 were prepared in ethylene glycol and added to RSK2 prior to thermal shift assay. Corresponding control experiments were carried out in the presence of 2.5% of ethylene glycol.

Dynamic light scattering (DLS) experiments were carried out at 10°C in a Dyna-Pro Temperature-controlled Microsampler (Wyatt Technology Corporation). RSK2 at ~0.7 mg/ml was analyzed in 50 mM Tris pH.8.0, 500 mM NaCl, 5mM 2-mercaptoethanol in the reaction volume of 25 μl. Samples were centrifuged for 10 minutes at 4°C and ~20000xg, immediately before measurements. Each sample was measured 20 times with 5-s acquisitions and the data were averaged. Polydispersity and hydrodynamic radius were calculated from fitting the autocorrelation data using the regularization method.

### Preparation of site-specific RSK2 mutants

The following RSK2 variants were generated, expressed and purified: S227A, T365A, S386A, T557A and K451A, T577A. All mutations were introduced using the QuickChange Site-Directed Mutagenesis Kit (Agilent Technologies) and confirmed by DNA sequencing. The respective protein variants were purified and activated as described above for the full-length wild type protein.

### Dephosphorylation of Ser386

RSK-2 protein (5μM) purified from *E*. *coli* was exposed to 400 U of lambda phosphatase (New England BioLabs® Inc.) in 1x NEBuffer for PMP (50mM Hepes pH 7.5, 100mM NaCl, 2mM DTT, 0.01% Brij 35) supplemented with 1mM MnCl_2_. Incubation was performed at RT for 30 minutes and checked using Western Blotting analysis.

### Phosphorylation of RSK2 protein in mouse blood vessels

All animal experiments were performed according to protocols reviewed and approved by the University of Virginia Institutional Animal Care and Use Committee and were in compliance with United States Public Health Service and Department of Agriculture guidelines for laboratory animal welfare. Five month old males C57Bl/J6 mice were euthanized and the abdominal aortae from the iliac branches to the region of the kidney were removed, placed in ice-cold HEPES-buffered Krebs solution, cleaned of their adventitia and equilibrated at 37°C in the HEPES-buffered Krebs solution for 1 hr. Aortas were incubated with MEK inhibitor U0126 (10μM) or PDK1 inhibitor GSK2334471 (10μM) or DMSO control for another hour followed by stimulation with TXA2 receptor mimetic U46619 (1μM) for 5 min and snap frozen in liquid N_2_. Protein phosphorylation was preserved by transferring the frozen tissue into 10% trichloroacetic acid in acetone at -80°C for freeze substitution, as described previously [40]. The trichloroacetic acid was removed by successive washes with pure acetone, after which the vessels were allowed to dry and then homogenized in sample buffer: 10 M Urea (1:1), and boiled for 5 min. After centrifugation at 10,000 × *g* for 10 min, the samples were subjected to PAGE electrophoresis and Western blotting.

## Results and Discussion

Although two previously published papers mention overexpression of full-length mammalian RSK2 kinase in *E*. *coli* as a fusion protein with a His_6_-tag [19, 37], the silver stained gels shown in those publications attest to the fact that yields were very low and purity was questionable. Nevertheless, we first attempted to overexpress the full-length murine RSK2 in *E*.*coli* with the N-terminal His_6_ tag and to purify this protein by affinity chromatography on Ni-NTA column using a linear gradient of imidazole for elution **(Figure A in S1 File)**. The resulting protein was degraded and/or heavily contaminated. Iterative use of the Ni-NTA column allowed for the preparation of a small amount of protein but after the removal of the His-tag by rTEV protease RSK2 could not be purified further because the protein bound strongly to the Ni-NTA resin **(Figure B in S1 File)**. Attempts to purify RSK2 on MonoQ or Phenyl Sepharose columns were unsuccessful. We concluded that the protocol is not viable for preparation of larger amounts of pure, homogeneous protein.

**Fig 2:**
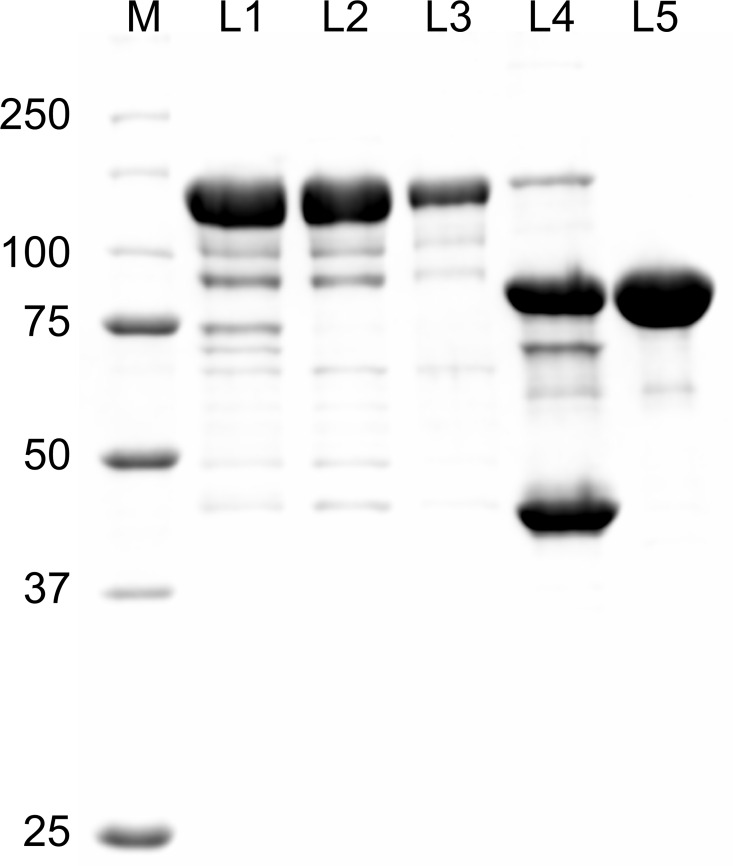
Purification of MBP-His_6_-RSK2. Proteins were separated by SDS-PAGE and stained using Coomassie Brilliant Blue. Lane 1, pooled fractions after Ni-NTA elution; lane 2, proteins eluted from amylose column; lane 3, proteins pooled fractions after second Ni-NTA elution; 4, proteins after digestion with TEV protease; lane 5, pooled fractions after purification by size exclusion chromatography.

We cloned the RSK2 gene into the pMBPHisParallel2 vector [39], to overexpress the kinase in fusion with the maltose binding protein (MBP), followed by a His_6_ tag and a cleavage site for TEV protease. The *E*. *coli* MBP is widely used as a fusion partner for target proteins that are difficult to express, often dramatically boosting yield and acting as a folding chaperone [41–43]. Because purification of the resulting fusion proteins using the amylose affinity chromatography is not always effective, it has been suggested that supplemental tags, such as the His_6_-tag can be used in conjunction with MBP, optimally placed at its C-terminus [42]. The MBP-His_6_-RSK2 fusion protein was overexpressed at high yield and, additionally, after purification on a Ni-NTA column showed significantly higher purity than the His_6_-tagged variant. Moreover, amylose resin efficiently separated the fusion protein from the remaining contaminants **(Fig 2)**. In contrast, direct purification of MBP-His_6_-RSK2 from crude lysates using amylose column, without prior Ni-NTA step, resulted in significantly lower yield (data not shown). The fusion protein eluted from the amylose column was re-applied onto Ni-NTA column, extensively washed to remove maltose bound to MBP, eluted with imidazole, digested with TEV protease, and passed through the amylose column again to remove MBP. The final purification step by size exclusion chromatography on Sephadex 200 yielded ~3 mg of purified RSK2 per liter of *E*.*coli* culture as determined by optical absorbance of the protein at 280 nm. When purified protein was analyzed by analytical size exclusion chromatography, it showed an asymmetric profile with a single peak roughly corresponding to a molecular weight of a monomeric RSK2 **(Figure B in S1 File).**

**Fig 3:**
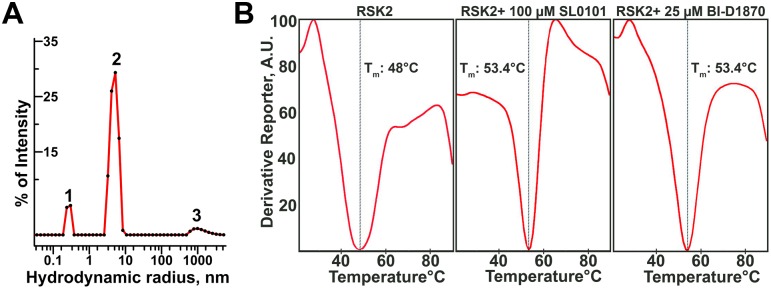
Biophysical characterization of RSK2. (A) Particle size distribution in the sample of RSK2 (0.7 mg/ml) as determined by DLS. Peak 2 accounts for 84% of sample by intensity, has a polydispersity of 24% and corresponds to a particle with an average molecular mass of 144 kDa. (B) Thermal stability assay of RSK2. The minimum (T_m_) of the derivative function of fluorescence intensity corresponds to a mid-point of unfolding (denaturation) curve of the protein.

We further evaluated the polydispersity and the molecular weight of the recombinant RSK2 using dynamic light scattering (DLS). We found that at 0.7 mg/ml the protein is predominantly a homodimer, with the hydrodynamic radius 5.0 nm of and a corresponding molecular weight of ~ 145 kDa **(Fig 3A)**. The polydispersity of the major peak was routinely found to be in the range of 23–25% indicating that protein is mostly dimeric with little or no contribution of monomers and higher oligomers to the light scattering signal. Thus, we conclude that RSK2 forms low affinity dimers especially in concentrated solutions (e.g. >0.5 mg/ml). Resolving RSK2 sample on a size exclusion column disrupts a large fraction of RSK2 dimers due to dilution, and shearing forces; nevertheless an asymmetric peak **(Figure B in S1 File)** is consistent with residual dimerization.

To ascertain proper folding of the protein, we used the thermal stability assay (TSA) to detect temperature-induced, denaturing transition [44]. We observed melting (unfolding) with the midpoint of transition at ~48°C **(Fig 3A)**. This was consistent with the protein being properly folded in solution. Furthermore, in the presence of BI-D1870 and SL0101, both of which are specific inhibitors of RSK2 [14, 45, 46], we observed an increase in the midpoint temperature of ~ 5°C consistent with proper binding of inhibitors and stabilization of the protein **(Fig 3B)**.

**Fig 4:**
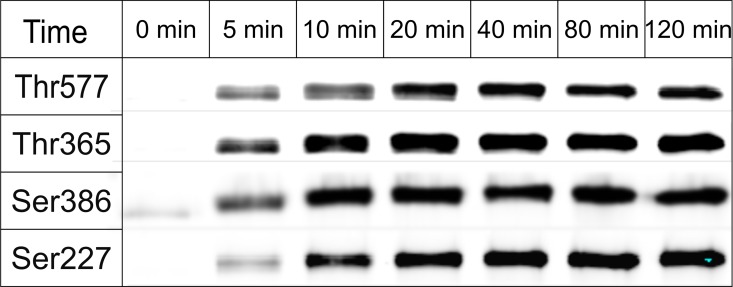
Time course of RSK2 phosphorylation by ERK2 and PDK1. Recombinant RSK2 was phosphorylated with the mixture of active PDK1 and ERK2. Equal aliquots of phosphorylation reaction were taken at indicated time points and subjected to immunoblot analysis using antibodies against specific phosphorylated sites (see methods).

**Fig 5:**
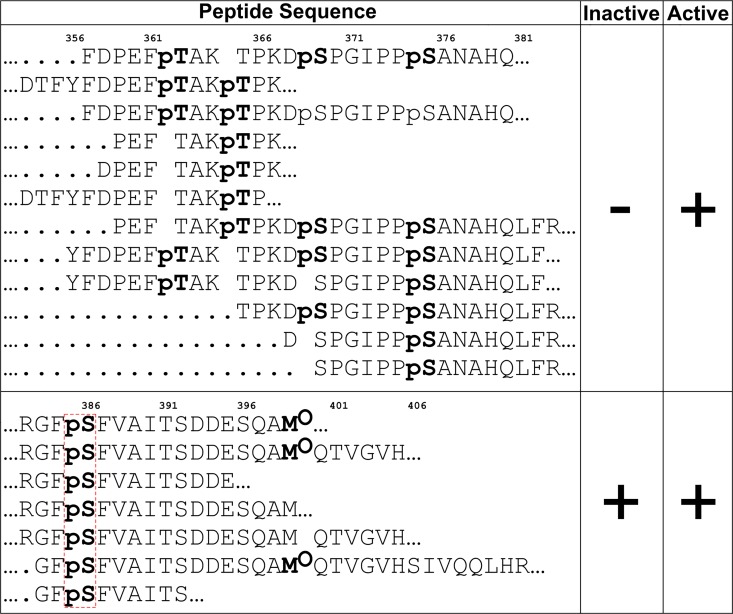
Select RSK2 phosphorylated peptides identified by mass spectrometry. Upper panel shows peptides from samples phosphorylated after incubation with ERK2 and PDK1, but not in nascent protein. Lower panel shows phosphorylated peptides in samples of nascent protein and after incubation with ERK2 and PDK1.

**Fig 6:**
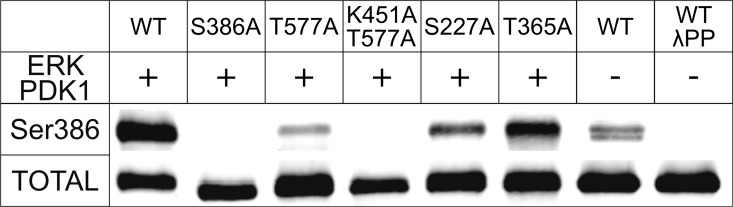
Western Blot detection of Ser386 phosphoprotein levels in wild-type and mutant RSK2. Samples of recombinant RSK2 were assayed for phosphorylation of Ser386 in the nascent form and after incubation with ERK2 and PDK1. The bottom panel shows the total amount of the protein identified by a generic antibody to RSK2 (see text for further details). The splitting of the band in the wild-type sample is occasionally observed likely due to heterogeneity of the sample–not affecting the key conclusion.

Next, we tested the susceptibility of recombinant RSK2 to *in vitro* phosphorylation at the canonical regulatory sites with activated recombinant ERK2 and commercial PDK1 kinases **(Fig 4).** The protein undergoes expected phosphorylation: Thr365, Thr577 and Ser386 are phosphorylated rapidly. Half-maximum phosphorylation levels were achieved within the first 5 minutes of the reaction. In contrast, Ser227 within the activation loop is phosphorylated at a lower rate. Interestingly, we observed low level phosphorylation on Ser386 even at 0 min **(Fig 4**). To ascertain if Ser386 is phosphorylated in the nascent protein, we carried out a mass spectrometry analysis of both nascent and phosphorylated samples. Several peptides containing Ser386 showed phosphorylation **(Fig 5).** While mass spectrometry is not a qualitative method, the result confirms that the western blot result is indeed due to partial phosphorylation, which we estimate at ~10%. In theory, phosphorylation could occur in *E*.*coli* due to endogenous kinases, but the fact that only Ser386 was phosphorylated hinted at the possibility that RSK2 may undergo autocatalytic phosphorylation. To verify this hypothesis, and to monitor possible interdependence between the phosphorylation events, we expressed and purified several variants of RSK2, i.e. T577A, K451A/T577A, T365A and S227A. Western blots using anti-pS386 antibodies **(Fig 6)** confirmed that the wild type protein is partly phosphorylated in its nascent state, and fully phosphorylated in the presence of ERK2 and PDK1. (We also carried out experiments with either ERK2 or PDK1 alone, with results fully consistent with the above experiments: the data are shown in **Figure D in S1 File**). As expected, the negative control (S386A) and the catalytically impaired K451A/T577A variants showed no phosphorylation (K451A mutation eliminates the catalytic residue from the active site of the CTKD). The T577A variant showed impaired phosphorylation on Ser386 in the presence of ERK2/PDK1, showing that the non-phosphorylated activation loop in CTKD impeded, but did not eliminate catalytic activity. The S227A variant was slightly less susceptible to phosphorylation on Ser386 compared to the wild type, and the T365A mutation had no effect.

The autophosphorylation of RSK2 on Ser386 is not completely unexpected. It has been reported, that upon the removal of the C-terminal α-helix, i.e. from residue 697, the CTKD of RSK2 becomes constitutively active in the absence of ERK [47]. Our work is the first demonstration of autophosphorylation using pure, full-length recombinant protein. This suggests, that *in vivo* there is a pool of Ser386-phosphorylated RSK2 that is activated by PDK1 alone; this pool may increase upon upregulation of RSK2 expression in pathological cases, e.g. in cancer cells.

Finally, mass spectrometry data for the phosphorylated sample revealed new target sites for ERK1/2 –Thr362 and Ser375. The phosphorylation of these sites has been previously reported *in vivo* by various proteomics studies (http://www.phosphosite.org), but their functional significance is not known.

Because our objective was to obtain fully dephosphorylated, inactive protein, we assessed the possibility of enzymatic removal or residual phosphorylation of pSer386 using protein phosphatase λ (ppλ), a potent enzyme with broad activity towards phosphoserine, phosphothreonine and phosphotyrosine. Indeed, ppλ was able to dephosphorylate Ser386 fully, as judged by western blots **(Fig 6).**

**Fig 7:**
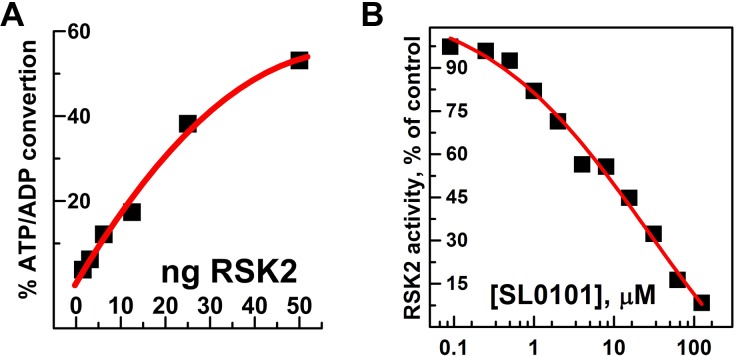
Enzymatic activity of RSK2 and its sensitivity to SL0101. **(A)** Different amounts of RSK2 (1.5–50 ng) were used to phosphorylate S6 peptide (0.2 mg/ml) in the presence of 100 μM ATP for 30 minutes at room temperature in the reaction volume of 5 μl. The percentage of ATP/ADP conversion was estimated from standard curve using ADP Glo assay. **(B)** The enzymatic activity of RSK2 (5 ng) was assessed at 10 μM of ATP in the presence of indicated concentrations of SL0101 and expressed as a percentage of RSK2 activity in the absence of inhibitor.

To complete the study, we assessed the enzymatic activity of the phosphorylated RSK2 using ADP-Glo™ kinase activity kit. This end-point assay allows a highly sensitive quantification of ATP consumed in the phosphorylation reaction. Using 0.2 mg/ml (0.16 mM) of S6 peptide as a substrate and 0.1 mM ATP we could easily detect conversion of ATP to ADP. Under conditions of low ng amounts of RSK2, the percentage of converted ATP was proportional to the amount of RSK2 in the reaction **(Fig 7A).** From these data we determined specific activity of RSK2 to be 258 nmol/min/mg, only slightly lower than 370 nmol/min/mg determined for fully activated human RSK2 purified from insect cells [45]. The higher nominal values of specific activity of commercial RSK2 from insect cells can be explained by higher temperature at which the kinase assay was carried out (30°C vs. 22°C in our studies). Phosphorylated RSK2 was also tested for sensitivity towards its inhibitor SL0101 [14, 46, 48]. Although relatively selective, SL0101 is not a very potent inhibitor of RSK2, therefore inhibition is usually assessed at low concentrations of ATP, usually 10 μM [15, 49]. The IC_50_ value for SL0101 is estimated to be in the low μM range [49, 50]. We observed a dose dependent inhibition of RSK2 activity by SL0101 with IC_50_ ~ 8 μM **(Fig 7B)**. This is in a good agreement with earlier studies that used RSK2 overexpressed in insect cells.

**Fig 8:**
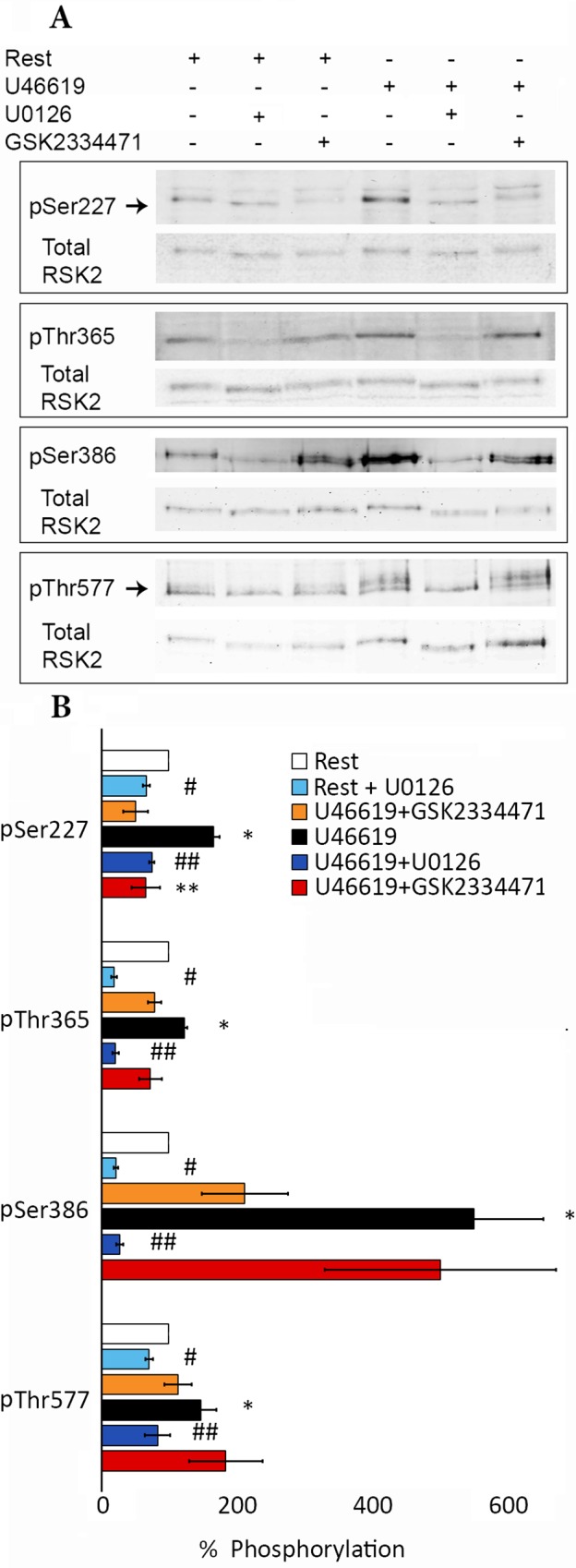
Phosphorylation of RSK2 *in vivo*. Densitometric quantification of RSK2 phosphorylation at Ser227, Thr365, Ser386 and Thr577 after 5 min stimulation with TXA2-analogue U46619 (1 μM) in the presence or absence of the MEK inhibitor U0126 (10 μM) or PDK1 inhibitor GSK2334471 (10μM) normalized to total RSK2 in mouse aortae. Arrows indicate the position of the total RSK2 band in the pSer227 and pThr577 immunoblots. The higher bands likely reflect other RSK isoforms not detected by the specific total RSK antibody, e.g. the anti-pSer227 RSK2 antibody is claimed to be broadly reactive with Ser227 phosphorylated Rsk family proteins and the anti-pThr577 antibody detects RSK1 and RSK 2 and these 2 isoforms differ by 2,099 amino acids in the mouse. **(A)** Western Blot analysis. **(B)** Bar-graph summary from three experiments. Rest values are taken as 1. Values shown are means ± S.E.M. * p<0.03, rest compared to U46619 stimulation; # p<0.02, rest compared to MEK inhibitor U0126 pretreatment; ## p<0.02, U46619 stimulation compared to MEK inhibitor U0126; * * p<0.03 U46619 stimulation compared to PDK inhibitor GSK2334471.

Finally, we explored the phosphorylation status of endogenous RSK2 in blood vessels before and after contraction induced by the thromboxane A2 analog U46619 **(Fig 8)**. Under resting conditions there is detectable phosphorylation of sites S227, T365, S386 and T577, that is significantly increased by U46619 stimulation in the absence but not presence of MEK inhibitors, in agreement with the scheme in **Fig 1**. In keeping with the above *in vitro* results on recombinant RSK, there was detectable phosphorylation at S386 even with inhibition of MEK/ERK/MAPK-induced phosphorylation under control as well as in the presence of U46619 supporting the concept of RSK susceptible to ERK-independent phosphorylation on Ser277. To ascertain that that the bands in our Western blots are indeed RSK phospho-Ser227 and phospho-Thr577 rather than non-specific labeling, we have also run an additional experiment in which we pre-absorbed the phospho-Ser227 and the phospho-Thr577 antibodies with phospho-Ser227 and phospho-Thr577 blocking peptides, which abolishes interaction **(Figure E in S1 File)**. Additionally evidence of antibody specificity is provided in **Figure E in S1 File** where phospho-Ser227 signal on Western blots is increased in the presence of serum stimulation of smooth muscle cells, suppressed in the presence of RSK inhibitors and the phospho-Ser227 inhibitor, GSK2334471 **(Figure E in S1 File)**. No signal is detected in smooth muscle cells from *RSK2-/-* mice **(Figure E in S1 File)**. Regarding ERK-independent phosphorylation on Ser277 there is evidence that Pyk2 kinase can phosphorylate and activate PDK2 independently of MEK [51]. Pyk2 has been shown to be present in smooth muscle and activated by stimulation [52, 53]. Thus, it is likely that the residual phospho-Ser227 reflects activity of kinases other than MEK, such as Pyk2. The residual phosphorylation on Ser386 creates a docking site for the Pyk2 activated PDK1.

In conclusion, we report a new, efficient method for production of wild-type, full length inactive RSK2 kinase in *E*. *coli*, yielding milligram quantities. The amount of protein and the level of purity are sufficient for virtually any kind of biophysical analysis. Bacterially produced RSK2 is unphosphorylated (except for trace phosphorylation of Ser386) and can be specifically and selectively phosphorylated *in vitro* by ERK and PDK1. A fully dephosphorylated and inactive RSK2 can be easily prepared by dephosphorylation in vitro using Lambda Protein Phosphatase. In contrast, RSK2 from eukaryotic hosts, e.g. insect cells, is expected to be phosphorylated on multiple sites, due to basal activity of kinases of ERK/MAPK pathway, resulting in a potentially heterogeneous sample. When fully activated, bacterially-expressed RSK2 is suitable for functional studies including high throughput screening of chemical libraries for drug discovery. The autocatalytic properties of RSK2 suggest that the protein undergoes slow modification on Ser386, making it susceptible to ERK-independent phosphorylation on Ser277. This has potential implications for signal transduction *in vivo*.

## Supporting Information

S1 File **Figure A. Purification of His-tagged RSK2**. (A) Optical absorption profile of His-tagged RSK2 eluted from Ni-NTA column and the analysis of collected fractions on Coomassie-stained SDS-PAGE gel. (B) Untagged RSK2 binds to Ni-NTA column. Proteins were separated by SDS-PAGE and stained by Coomassie brilliant blue. Pooled fractions after second purification of RSK2 on Ni-NTA were visualized before (lane 1) and after (lane 2) digestion with TEV protease to remove the His-tag. Untagged RSK2 was passed through the Ni-NTA column: lanes 3, 4 and 5 show proteins in unbound, wash and bound fractions respectively. The fact that untagged RSK2 binds to Ni-NTA was also verified by using the protein obtained after the removal of MBP-(His)_6_-tag (data not shown). **Figure B. Analytical size-exclusion of purified RSK2**. A 0.4 ml sample of RSK2 (0.6 mg/ml) was resolved on Superdex 200 column. Five 1 ml fractions with positive optical absorbance at 280 nm were collected and analyzed on Coomassie-stained polyacrylamide gel. Approximate positions of molecular weight standards are shown as arrows in the upper part of the figure. An asymmetric nature of the peak is likely to be due to a weak dimerization of RSK2. **Figure C**. **Thermal stability of nascent and activated RSK2**. Purifed non-phosphorylated (A) and in vitro phosphorylated (B) RSK2 were assessed for thermostability using thermofluor assay described in Materials and Methods. **Figure D. Western Blot detection of phosphoprotein levels in wild-type and mutant RSK2 by ERK or PDK1.** Samples of recombinant RSK2 were assayed for phosphorylation of Ser386 in the nascent form and after incubation with ERK2 or PDK1. The bottom panel shows the total amount of the protein identified by a generic antibody to RSK2 (see text for further details). The splitting of the bands is occasionally observed due to heterogeneity of the sample; each row shows a single membrane. **Figure E. Demonstration of specificity of the antibody used for detection of phosphorylated RSK2 at the Ser227 site.** A) Western blot of serial dilutions of mouse abdominal aorta lysate incubated with anti-pSer227 or anti-pThr577 RSK2 antibodies (upper panels) or with anti-pSer227 or anti-pThr577 RSK2 antibodies pre-absorbed with blocking peptides of the same sequences used to generate the antibodies (lower panels). Tubulin bands indicate protein loading for each lane. pSer227 RSK2 antibody, sc-12445R, blocking peptide, sc-12445P, pThr 577 RSK2 antibody, sc-16407R, blocking peptide, sc-16407P, Santa Cruz. B) Western blots of mouse aortic smooth muscle cells from *Rsk2-/-* and wild type mice stimulated with serum and pre-incubated with RSK2 inhibitors LJH685 (1μM) plus LJI308 (1μM) or the PDK1 inhibitor GSK2334471 (1 μM). Cells were starved overnight and treated with inhibitors for 1hr followed by stimulation with 5% fetal serum for 5 min. Following electrophoresis, membranes were reacted with anti-pSer227 RSK2 antibody or anti-tubulin antibody as a protein loading control. Note that serum stimulation increases phosphorylation at Ser227, indicative of RSK2 activation and that this is markedly suppressed in the presence of LJH685/LJI308 and GSK2334471 (upper panel) and that in the absence of RSK2 no band is detected (lower panel), indicative of the specificity of the anti-pSer227 RSK2 antibody used in this study.
(DOCX)
