# Supplementary material for: Bacterial Expression, Purification and In Vitro Phosphorylation of Full-Length Ribosomal S6 Kinase 2 (RSK2)
Source: PLoS One. 2016 Oct 12;11(10):e0164343. doi: 10.1371/journal.pone.0164343 (PMC5061434; doi:10.1371/journal.pone.0164343)
Supplement: S1 File — Figure A. Purification of His-tagged RSK2. (A) Optical absorption profile of His-tagged RSK2 eluted from Ni-NTA column and the analysis of collected fractions on Coomassie-stained SDS-PAGE gel. (B) Untagged RSK2 binds to Ni-NTA column. Proteins were separated by SDS-PAGE and stained by Coomassie brilliant blue. Pooled fractions after second purification of RSK2 on Ni-NTA were visualized before (lane 1) and after (lane 2) digestion with TEV protease to remove the His-tag. Untagged RSK2 was passed through the Ni-NTA column: lanes 3, 4 and 5 show proteins in unbound, wash and bound fractions respectively. The fact that untagged RSK2 binds to Ni-NTA was also verified by using the protein obtained after the removal of MBP-(His)6-tag (data not shown). Figure B. Analytical size-exclusion of purified RSK2. A 0.4 ml sample of RSK2 (0.6 mg/ml) was resolved on Superdex 200 column. Five 1 ml fractions with positive optical absorbance at 280 nm were collected and analyzed on Coomassie-stained polyacrylamide gel. Approximate positions of molecular weight standards are shown as arrows in the upper part of the figure. An asymmetric nature of the peak is likely to be due to a weak dimerization of RSK2. Figure C. Thermal stability of nascent and activated RSK2. Purifed non-phosphorylated (A) and in vitro phosphorylated (B) RSK2 were assessed for thermostability using thermofluor assay described in Materials and Methods. Figure D. Western Blot detection of phosphoprotein levels in wild-type and mutant RSK2 by ERK or PDK1. Samples of recombinant RSK2 were assayed for phosphorylation of Ser386 in the nascent form and after incubation with ERK2 or PDK1. The bottom panel shows the total amount of the protein identified by a generic antibody to RSK2 (see text for further details). The splitting of the bands is occasionally observed due to heterogeneity of the sample; each row shows a single membrane. Figure E. Demonstration of specificity of the antibody used for detection of phosphorylate [file pone.0164343.s001.docx]

**Supplementary information (Utepbergenov et al.)**


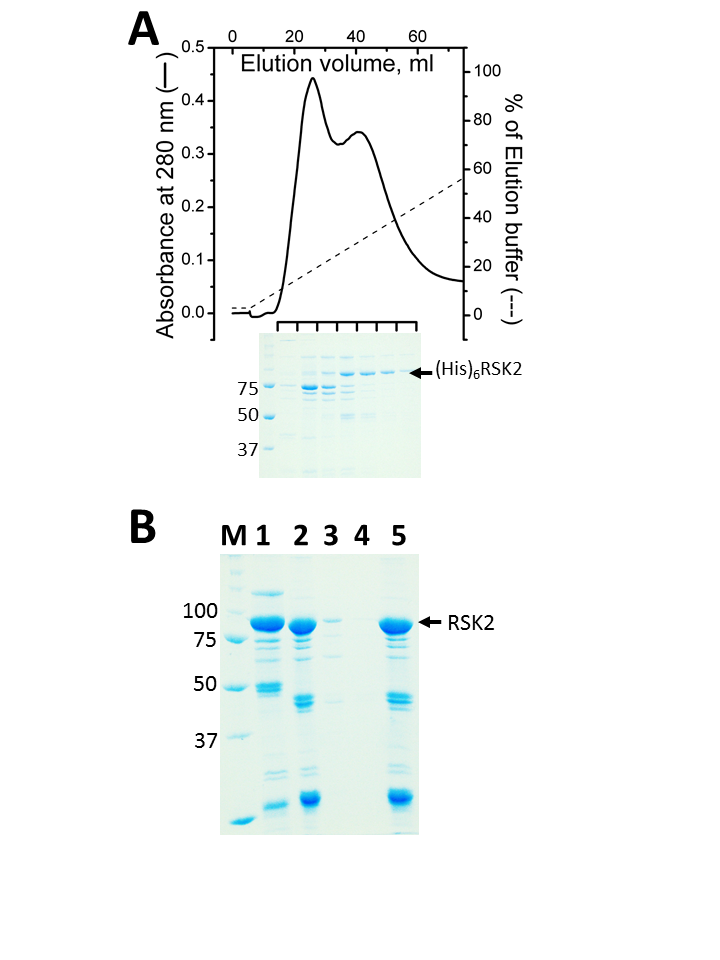


**Figure A.** **Purification of His-tagged RSK2**. (A) Optical absorption profile of His-tagged RSK2 eluted from Ni-NTA column and the analysis of collected fractions on Coomassie-stained SDS-PAGE gel. (B) Untagged RSK2 binds to Ni-NTA column. Proteins were separated by SDS-PAGE and stained by Coomassie brilliant blue. Pooled fractions after second purification of RSK2 on Ni-NTA were visualized before (lane 1) and after (lane 2) digestion with TEV protease to remove the His-tag. Untagged RSK2 was passed through the Ni-NTA column: lanes 3, 4 and 5 show proteins in unbound, wash and bound fractions respectively. The fact that untagged RSK2 binds to Ni-NTA was also verified by using the protein obtained after the removal of MBP-(His)_6_-tag (data not shown).


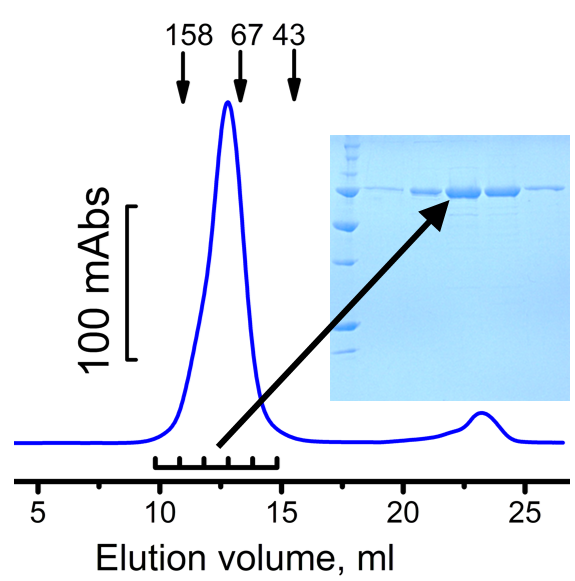


**Figure B.** **Analytical size-exclusion of purified RSK2**. A 0.4 ml sample of RSK2 (0.6 mg/ml) was resolved on Superdex 200 column. Five 1 ml fractions with positive optical absorbance at 280 nm were collected and analyzed on Coomassie-stained polyacrylamide gel. Approximate positions of molecular weight standards are shown as arrows in the upper part of the figure. An asymmetric nature of the peak is likely to be due to a weak dimerization of RSK2.


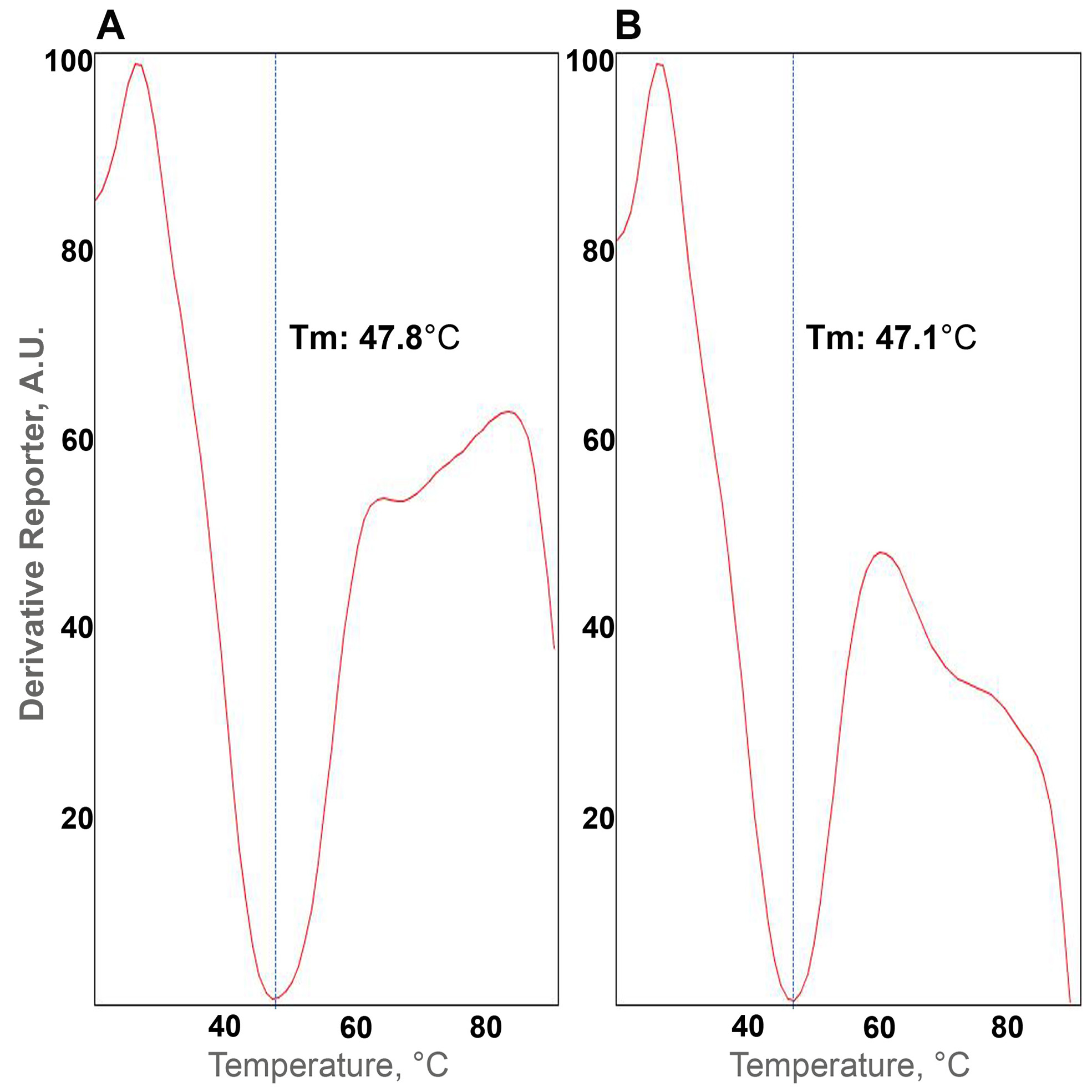


**Figure C**. **Thermal stability of nascent and activated RSK2**. Purifed non-phosphorylated (A) and in vitro phosphorylated (B) RSK2 were assessed for thermostability using thermofluor assay described in Materials and Methods.


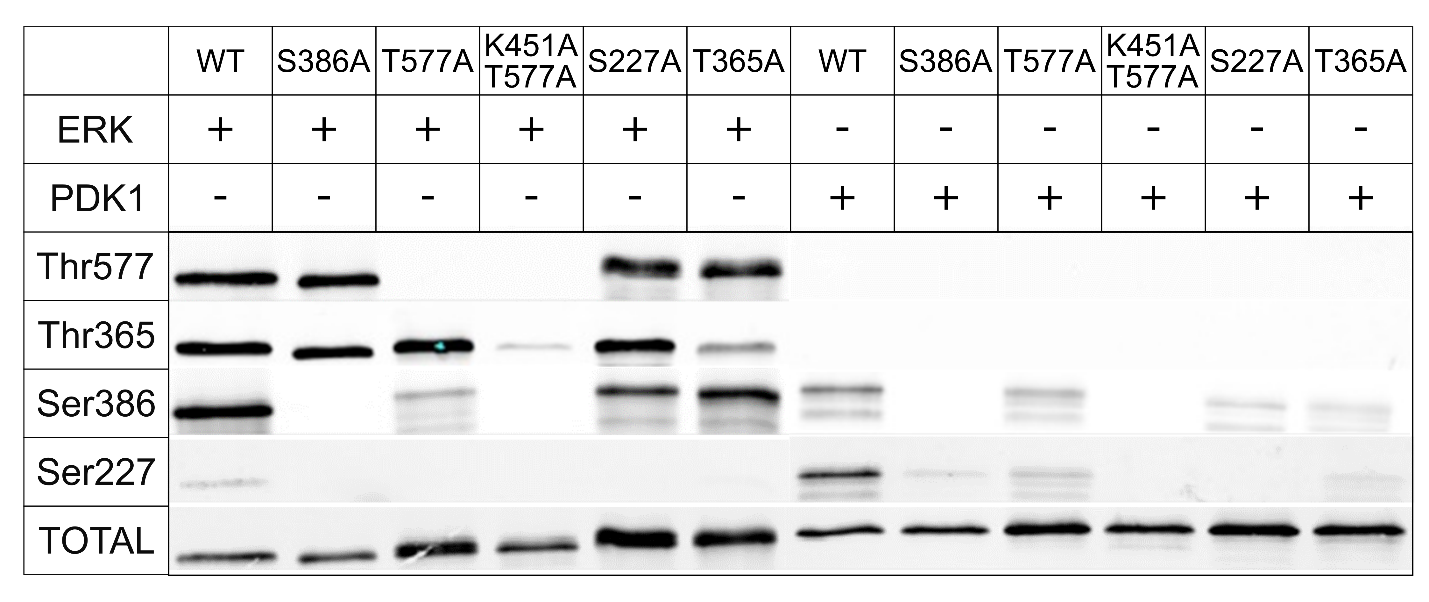


**Figure D.** **Western Blot detection of phosphoprotein levels in wild-type and mutant RSK2 by ERK or PDK1.**  Samples of recombinant RSK2 were assayed for phosphorylation of Ser386 in the nascent form and after incubation with ERK2 or PDK1. The bottom panel shows the total amount of the protein identified by a generic antibody to RSK2 (see text for further details). The splitting of the bands is occasionally observed due to heterogeneity of the sample; each row shows a single membrane.


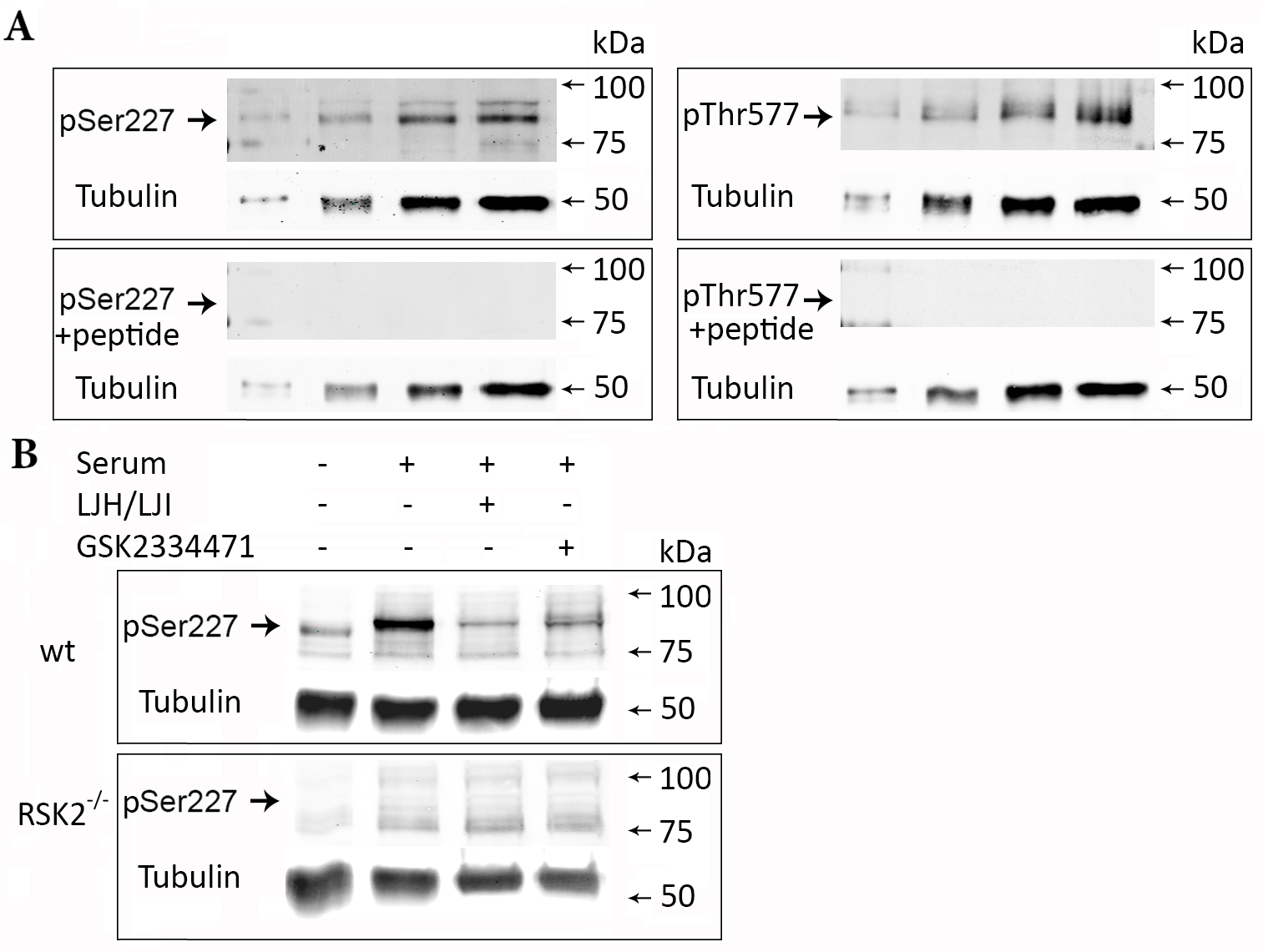


**Figure E. Demonstration of specificity of the antibody used for detection of phosphorylated RSK2 at the Ser227 site.** A) Western blot of serial dilutions of mouse abdominal aorta lysate incubated with anti-pSer227 or anti-pThr577 RSK2 antibodies (upper panels) or with anti-pSer227 or anti-pThr577 RSK2 antibodies pre-absorbed with blocking peptides of the same sequences used to generate the antibodies (lower panels). Tubulin bands indicate protein loading for each lane. pSer227 RSK2 antibody, sc-12445R, blocking peptide, sc-12445P, pThr 577 RSK2 antibody, sc-16407R, blocking peptide, sc-16407P, Santa Cruz. B) Western blots of mouse aortic smooth muscle cells from *Rsk2-/-* and wild type mice stimulated with serum and pre-incubated with RSK2 inhibitors LJH685 (1μM) plus LJI308 (1μM) or the PDK1 inhibitor GSK2334471 (1μM). Cells were starved overnight and treated with inhibitors for 1hr followed by stimulation with 5 % fetal serum for 5 min. Following electrophoresis, membranes were reacted with anti-pSer227 RSK2 antibody or anti-tubulin antibody as a protein loading control. Note that serum stimulation increases phosphorylation at Ser227, indicative of RSK2 activation and that this is markedly suppressed in the presence of LJH685/LJI308 and GSK2334471 (upper panel) and that in the absence of RSK2 no band is detected (lower panel), indicative of the specificity of the anti-pSer227 RSK2 antibody used in this study.
